# Brain tissue oxygen monitoring for severe traumatic brain injury: the international multicentre randomised controlled BONANZA-GT study protocol

**DOI:** 10.1136/bmjopen-2025-106962

**Published:** 2025-10-02

**Authors:** Andrew Alexander Udy, Toby Jeffcote, Camila R Battistuzzo, Aideen Sharry, Alexios A Adamides, Kate Ainscough, Patricia V Alliegro, James Anstey, Michael Bailey, Jesús Abelardo Barea-Mendoza, Judith Bellapart, Pierre Bouzat, Kathleen Byrne, Anthony Delaney, Katharine J Drummond, Matthias Haenggi, Leanne M Hays, Alisa Higgins, Olivier Huet, Martin K Hunn, Stephan M Jakob, Philip M Lewis, Kuan-Ying Lu, David Menon, James Moore, Patrick T Murray, John Porter, Benjamin Reddi, Jeffrey V Rosenfeld, Jonathon D Taylor, Anthony Trapani, Shirley Vallance, Stefan Wolf, Jean-Francois Payen, Fabio Silvio Taccone, Alistair Nichol, D James Cooper

**Affiliations:** 1 Australian and New Zealand Intensive Care Research Centre, Monash University, Melbourne, Victoria, Australia; 2 Department of Intensive Care and Hyperbaric Medicine, Alfred Hospital, Melbourne, Victoria, Australia; 3 Clinical Research Centre, University College Dublin, Dublin, Ireland; 4 Department of Neurosurgery, The Royal Melbourne Hospital, Melbourne, Victoria, Australia; 5 Department of Intensive Care, The Royal Melbourne Hospital, Parkville, Victoria, Australia; 6 Intensive Care Unit, Hospital Universitario 12 de Octubre, Madrid, Comunidad de Madrid, Spain; 7 Royal Brisbane and Women’s Hospital, Metro North Health, Queensland, Australia; 8 Department of Anaesthesia and Intensive Care, Centre Hospitalier Universitaire de Grenoble, Grenoble, France; 9 Critical Care Program, The George Institute for Global Health, Sydney, New South Wales, Australia; 10 Institute of Intensive Care Medicine, University Hospital Zurich, Zürich, Switzerland; 11 Department of Anaesthesia and Critical Care, Brest University Hospital Centre, Brest, Brittany, France; 12 Department of Neurosurgery, Alfred Hospital, Melbourne, Victoria, Australia; 13 University of Bern, Bern, Switzerland; 14 Department of Surgery, Monash University, Melbourne, Victoria, Australia; 15 Department of Medicine, University of Cambridge, Cambridge, UK; 16 Intensive Care Unit, Wellington Hospital, Wellington, New Zealand; 17 School of Medicine, University College Dublin, Dublin, Ireland; 18 Intensive Care Unit, University Hospitals Sussex NHS Foundation Trust, Worthing, UK; 19 Intensive Care Unit, Royal Adelaide Hospital, Adelaide, South Australia, Australia; 20 Department of Critical Care Medicine, Auckland City Hospital, Auckland, New Zealand; 21 Department of Neurosurgery, Charité - Universitätsmedizin Berlin, Berlin, Germany; 22 Department of Intensive Care, Hôpital Universitaire de Bruxelles (HUB), Université Libre de Bruxelles (ULB), Bruxelles, Belgium

**Keywords:** Surgery, Intensive Care, Traumatic Brain Injury, Intracranial pressure, Brain hypoxia

## Abstract

**Introduction:**

The management of severe traumatic brain injury (sTBI) in the intensive care unit (ICU) is focused on preventing secondary brain insults, by ensuring adequate cerebral perfusion, oxygenation and substrate delivery. Despite optimisation of intracranial pressure (ICP) and cerebral perfusion pressure (CPP) using evidence-based guidelines, brain tissue hypoxia can still occur and is strongly associated with adverse functional outcomes post sTBI.

**Methods and analysis:**

The Brain Oxygen Neuromonitoring in Australia and New Zealand Assessment – Global Trial (BONANZA-GT) is an international, two-arm, open-label, parallel group, randomised controlled trial comparing sTBI management incorporating early brain tissue oxygen (PbtO_2_) monitoring and optimisation, with ICP/CPP-based management alone. A total of 860 adults admitted to participating institutions with non-penetrating sTBI and requiring insertion of an ICP monitor (as determined by the treating neurosurgeon) will be enrolled. The primary outcome is the proportion of patients with favourable neurological outcomes, as defined by a Glasgow Outcome Score-Extended (GOS-E) >4, at 6 months following injury. Key secondary outcomes include all-cause mortality at ICU discharge, hospital discharge, adverse events, as well as hospital and ICU length of stay and GOS-E at 12 months. The BONANZA-GT will determine whether a protocolised therapeutic strategy guided by continuous PbtO_2_ monitoring in addition to ICP/CPP targets results in improved neurological outcomes when compared with standard care using ICP/CPP-guided management alone.

**Ethics and dissemination:**

Approval has been obtained from relevant ethics boards in every jurisdiction that is participating in the trial. Inclusion of adults who lack capacity for informed consent will be governed in accordance with the legal requirements of each participating site. Study findings will be presented at scientific meetings and disseminated via peer-review publications.

**Trial registration number:**

Australian and New Zealand Clinical Trials Registry (ACTRN 12619001328167).

**STRENGTHS AND LIMITATIONS OF THIS STUDY**

- The Brain Oxygen Neuromonitoring in Australia and New Zealand Assessment – Global Trial (BONANZA-GT) is an international multicentre, two-arm, open-label, parallel group, randomised controlled trial involving 10 different countries.
- The study will determine whether early and continuous brain tissue oxygen (PbtO_2_) monitoring and treatment in addition to intracranial pressure/cerebral perfusion pressure management improves functional recovery of patients with severe traumatic brain injury (sTBI), compared with standard care alone.
- The trial is sufficiently powered to detect 10% absolute difference in the primary outcome (Glasgow Outcome Scale-Extended at 6 months post injury) between the control and intervention groups.
- Findings may have limited application to the care of sTBI patients in less well-resourced healthcare systems.
- BONANZA-GT will provide important and greatly needed evidence to support or refute the role of PbtO_2_ monitoring in the acute care of patients suffering sTBI.

## Introduction

 Severe traumatic brain injury (sTBI) is a leading cause of death and disability in young adults and remains a global public health problem.^1 2^ Survivors often suffer long-term neurological disability, with only half able to live independently 6 months after their injury.^2^ Given that many sTBI patients suffer lifelong disability, the personal, social and economic costs of sTBI are unacceptably high.

Current acute management is focused on the surgical evacuation of space-occupying lesions and the avoidance of clinical factors known to exacerbate secondary injury (eg, hypotension, hypoxia and intracranial hypertension). Although high-quality prospective evidence for intracranial pressure (ICP) monitoring is lacking, data suggest protocolised interventions to lower ICP can improve outcomes after TBI.^3^ As a result, neurointensive care management of sTBI is primarily based on the achievement of guideline-based cerebral perfusion pressure (CPP) and ICP targets.^4 5^

However, there is a large body of histopathological,^6^ imaging^7^ and cerebral microdialysis^8 9^ data demonstrating the occurrence of brain tissue hypoxia following sTBI. Tissue hypoxia following sTBI is multifactorial and occurs due to capillary transit time heterogeneity, tissue compression^10^ and ischaemia secondary to insufficient CPP.^11^ There is also a complex interplay between tissue hypoxia, dysoxia and mitochondrial dysfunction that occurs amid high cerebral metabolic demand and limited oxygen reserve.^12^ Observational studies suggest that brain tissue oxygen tension (PbtO_2_) below a critical threshold is associated with unfavourable neurologic outcomes. Bardt *et al* found that the OR of severe disability or death was 10.8 (95% CI 2.09 to 55.7, p=0.004) for patients with PbtO_2_ values <10 mmHg for >30 min,^13^ a finding supported by other similar studies.^14 15^

These findings underscore the potential benefit of monitoring for brain tissue hypoxia and the implementation of clinical management that optimises PbtO_2_. Moreover, early clinical research has demonstrated the feasibility of protocolised interventions to increase PbtO_2_.^16^ The Brain Oxygen Optimisation in Severe TBI phase II (BOOST-2) study (n=119) showed a signal towards reduced mortality and increased favourable neurological outcomes in sTBI patients receiving PbtO_2_-guided interventions in addition to ICP/CPP-guided therapy.^17^ Conversely, a phase III multicentre randomised controlled trial (Oxy-CT, n=318) demonstrated no significant benefit from PbtO_2_ optimisation on neurological recovery.^18^

Given the ongoing uncertainty surrounding the benefits of PbtO_2_ optimisation in sTBI, the primary objective of the Brain Oxygen Neuromonitoring in Australia and New Zealand Assessment – Global Trial (BONANZA-GT) is to determine whether a neurointensive care protocol guided by continuous PbtO_2_ monitoring in addition to ICP/CPP targets compared with standard care using ICP/CPP targeted management alone, improves independent functional and neurological outcomes 6 months after injury.

## Methods and analysis

### Trial design

BONANZA-GT is an international, multicentre, two-arm, open-label, randomised controlled trial of early PbtO_2_ monitoring and optimisation in patients with sTBI. This trial has been registered with the Australian and New Zealand Clinical Trials Registry (ACTRN 12619001328167). Recruitment commenced on the 13 January 2020 and is expected to be completed by December 2028.

#### Patient and public involvement

Patients and/or the public were not involved in the design, or conduct, or reporting, or dissemination plans of this research.

**Figure 1:**
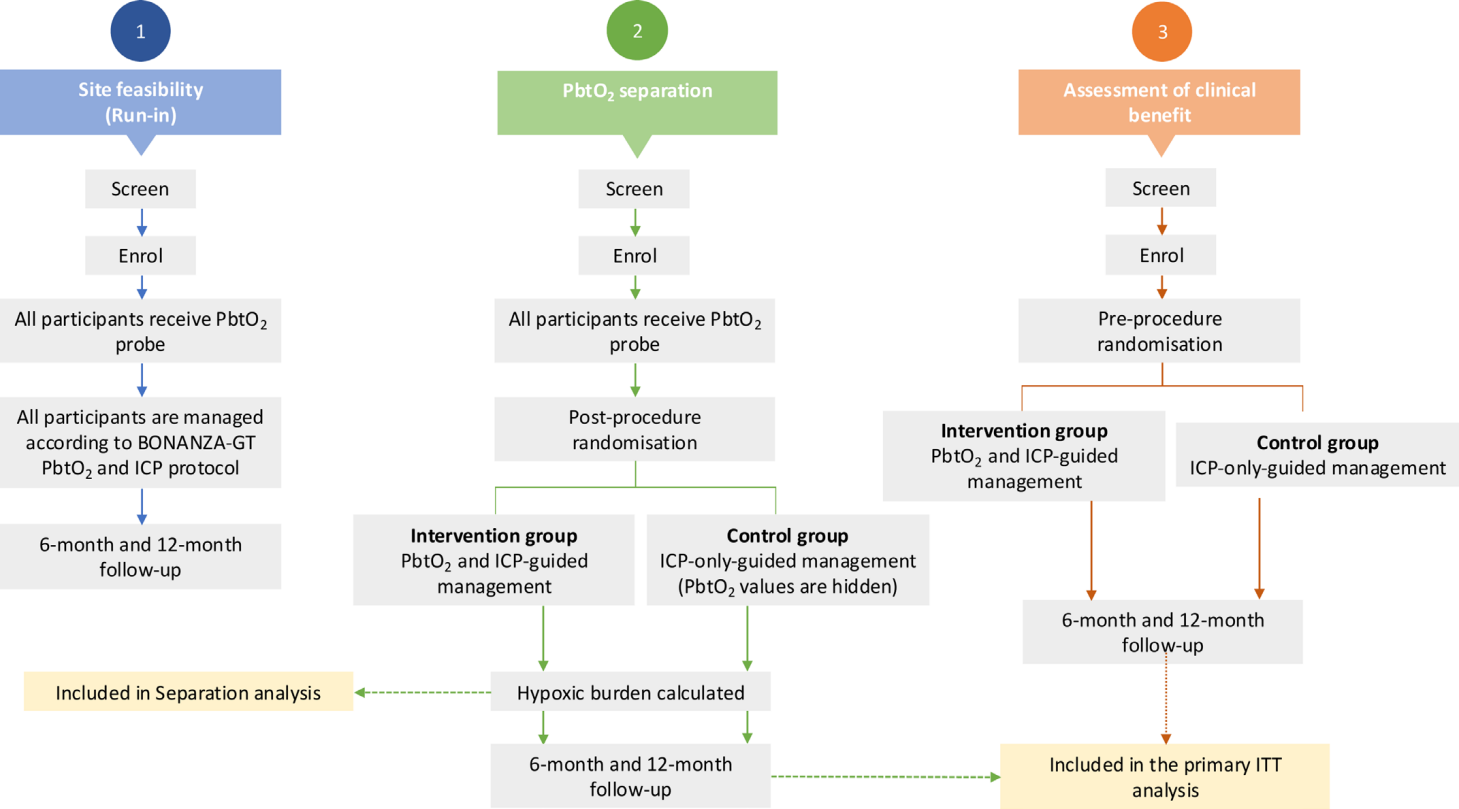
Outlining the three different phases of BONANZA-GT. BONANZA-GT, Brain Oxygen Neuromonitoring in Australia and New Zealand Assessment – Global Trial; ICP, intracranial pressure; ITT, intention-to-treat; PbtO_2_, brain tissue oxygen.

The trial consists of three phases as detailed in figure 1.

#### Phase I

At trial commencement, each new participating site has the option of completing a *run-in phase* if permissible under local ethics regulations. This involves the enrolment of up to four non-randomised patients, all of whom receive PbtO_2_ probes and are managed according to the PbtO_2_/ICP optimisation algorithm. This phase allows sites to resolve any unforeseen logistical, technical or clinical issues with the PbtO_2_ probe and the PbtO_2_ optimisation algorithm. These patients are additional and will not be included in the primary analysis, although they do contribute safety data.

#### Phase II

**Figure 2:**
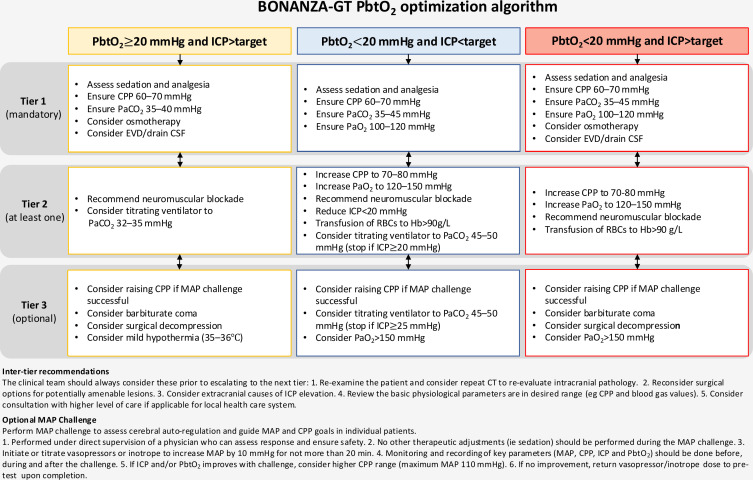
Detailed description of the Brain Oxygen Neuromonitoring in Australia and New Zealand Assessment PtbO_2_ optimisation algorithm for the management of patients allocated to the intervention arm. The PbtO_2_ treatment algorithm is a set of physiological interventions that vary depending on the clinical scenario. The physiological interventions are tiered in a hierarchical fashion, with less aggressive interventions attempted before aggressive manoeuvres. CPP, cerebral perfusion pressure; CSF, cerebrospinal fluid; EVD, external ventricular drain; Hb, haemoglobin; ICP, intracranial pressure; MAP, mean arterial pressure; PaCO_2_, partial arterial pressure of carbon dioxide; PaO_2_, partial arterial pressure of oxygen; PbtO_2_, brain tissue oxygen.

**Figure 3:**
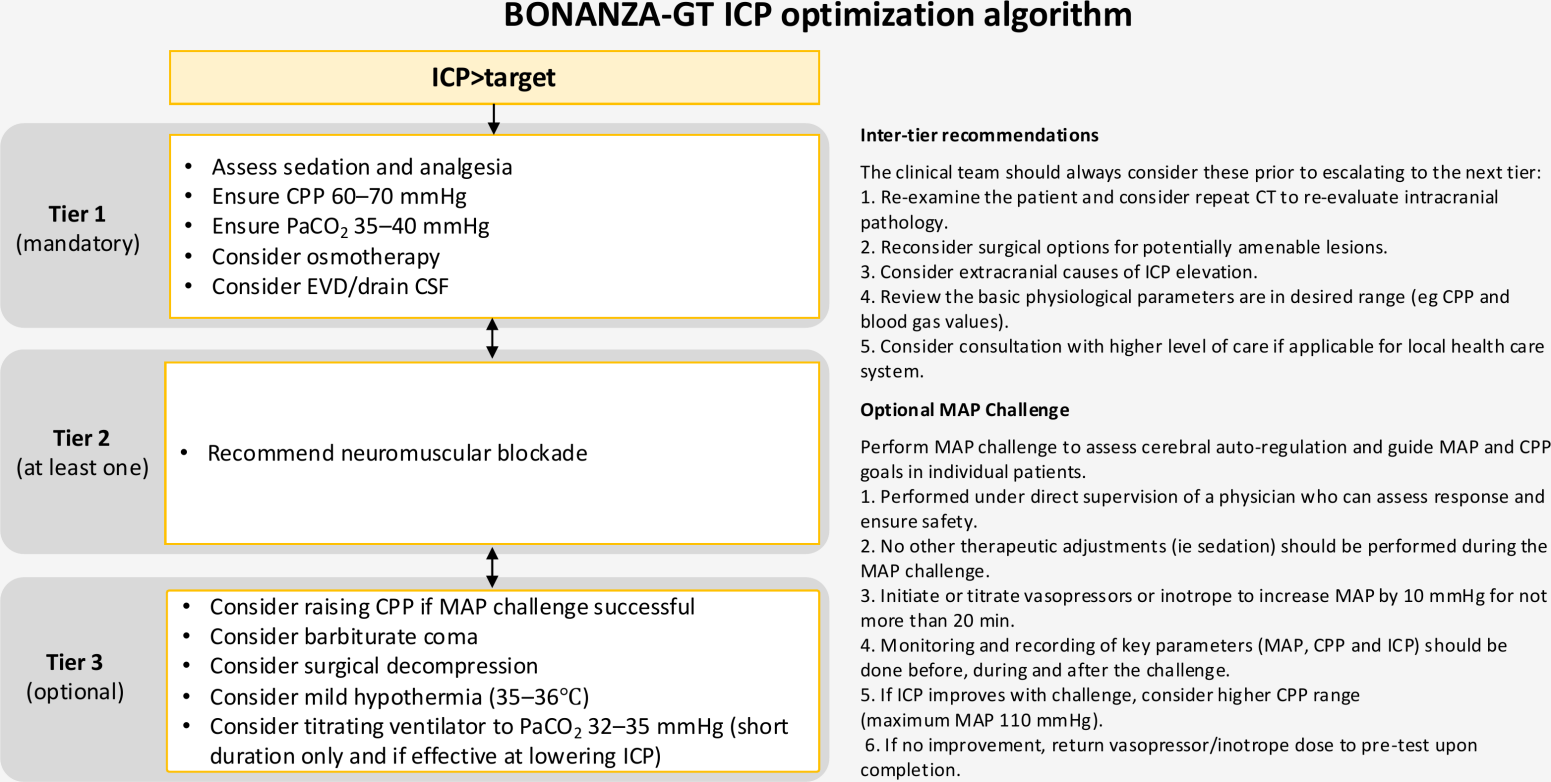
Detailed description of the Brain Oxygen Neuromonitoring in Australia and New Zealand Assessment ICP optimisation algorithm for the management of patients allocated to the control arm and receiving ICP-driven interventions alone. CPP, cerebral perfusion pressure; CSF, cerebrospinal fluid; EVD, external ventricular drain; ICP, intracranial pressure; MAP, mean arterial pressure; PaCO_2_, partial arterial pressure of carbon dioxide.

To demonstrate separation in PbtO_2_ values between the two groups, all patients (control and intervention arms) had both PbtO_2_ and ICP catheters inserted and secured via a dual channel Licox PMO bolt (Integra LifeSciences, Plainsboro, NJ, USA) or other locally approved techniques. Randomisation occurred post-procedure. Following randomisation and device calibration, bedside clinical staff were blinded to PbtO_2_ values in control patients with the placement of a locked cover over the Licox monitor. Patients allocated to the intervention arm were treated according to the integrated BONANZA-GT PbtO_2_/ICP optimisation algorithm (figure 2). Patients allocated to the control arm received standard care (according to ICP-based interventions alone, figure 3). Control group PbtO_2_ values were only accessible to unblinded study coordinators for periodic checks of appropriate probe response. Enrolment into phase I was completed on 15 December 2023 following the conclusion of a planned treatment separation analysis. The findings from phase II will be reported separately.

#### Phase III

Patients enrolled into phase III will be randomised to either standard care (ICP-only-guided management) or integrated PbtO_2_/ICP optimisation, prior to device insertion. Patients randomised to the intervention arm will have ICP and PbtO_2_ monitoring devices inserted as per local practice; no PbtO_2_ catheter will be inserted in control patients. Patients allocated to the intervention arm will be treated according to the integrated BONANZA-GT PbtO_2_/ICP optimisation algorithm (figure 2). Patients randomised to the control arm will be treated according to the ICP/CPP optimisation algorithm (figure 3). Placement of intracranial monitoring devices must occur as early as possible after randomisation.

### Study setting

BONANZA-GT involves multiple participating neurotrauma centres across Australia, Belgium, Finland, France, Germany, Ireland, New Zealand, Spain, Switzerland and the United Kingdom.

### Study population

Adult patients (≥18 years) admitted to a participating institution with a non-penetrating sTBI who require insertion of ICP monitoring are eligible for enrolment into BONANZA-GT. Details of the eligibility criteria are summarised in Box 1.

Box 1 **Detailed description of Brain Oxygen Neuromonitoring in Australia and New Zealand Assessment – Global Trial inclusion and exclusion criteria**

**Inclusion criteria**

1. Non-penetrating sTBI, as defined by:
  - A post-resuscitation GCS of 3–8 (motor sub-score <6) AND an abnormal CT scan (demonstrating intracranial trauma), OR
  - A post-resuscitation GCS of 3–8 (motor sub-score <6), in the absence of confounders, AND a high index of suspicion for sTBI (in the opinion of the treating neurosurgeon).
2. ≥18 years old.
3. There is a requirement for ICP monitoring (as determined by the treating neurosurgeon).

**Exclusion criteria**

1. Contraindication to placement of ICP and/or PbtO_2_ monitor (un-correctable coagulopathy).
2. Intracranial monitoring is already in situ at the time of neurosurgical assessment.
3. At the time of the decision to insert an ICP monitor, 12 hours or more had elapsed since the injury occurred.
4. Pregnancy.
5. Bilaterally absent pupillary response (eg, both pupils must be visible and are fixed and dilated) in the absence of confounders (eg, traumatic mydriasis, atropine and neuromuscular blockers).
6. Characteristics that preclude accurate optimisation of PbtO_2_:
  - Refractory hypotension (eg, systolic blood pressure <90 mmHg despite medical intervention).
  - Refractory systemic hypoxia (eg, arterial oxygen saturation <90% despite medical intervention).
  - Active CNS disease that is likely to impair accurate PbtO_2_ measurements (prior TBI and active CNS disease in vicinity of probe position).
7. Non-survivable injury or no intention of aggressive intervention.
8. Cardiac arrest as part of this traumatic event.
9. Other issues prior to randomisation that would preclude appropriate treatment, diagnosis or follow-up, such as known pre-existing neurological disease with confounding residual deficits or known pre-existing condition resulting in an inability to perform activities of daily living without assistance.

CNS, central nervous system, GCS, Glasgow Coma Scale; ICP, intracranial pressure, PbtO_2_, brain tissue oxygen; sTBI, severe traumatic brain injury.

### Randomisation and blinding

Trial patients will be recruited from the emergency department, operating theatre or intensive care unit (ICU) at participating sites. Patients will be assessed by trained study staff and, if eligible, randomised via a centralised password protected web-based system that conceals the random allocation sequence. Given that patients will lack the ability to provide informed consent at the time of recruitment, inclusion in the study will be on the basis of a deferred consent model (see Ethics and dissemination section for further details).

Randomisation will be stratified by study site and in permuted blocks of variable size. A patient is considered randomised when the treatment allocation is generated. All patients enrolled in the study will be followed until the end of study (12 months post-injury), until death or until consent is withdrawn. All enrolled patients (phase II and III) will be included in the primary intention-to-treat analysis. The 6-month and 12 month follow-up outcome assessments will be performed by trained central assessors who are blinded to group allocation.

### PbtO_2_ probe insertion

For phase III of the study, all participating sites have the option of using any registered, commercially available and approved PbtO_2_ monitor. The monitoring devices are stored according to the manufacturer’s instructions prior to insertion. Placement should occur within 24 hours of the time of injury, and failure to achieve this will constitute a protocol violation.

The treating team will exercise clinical judgement in selecting the site of PbtO_2_ catheter insertion, but should adhere to the following principles:

1. In intervention patients, combined ICP and PbtO_2_ monitoring should ideally be placed at the same time via a single burr hole in the operating theatre or at the bedside. This will minimise procedure time and obviate the need for a second burr hole. Variations on this technique are permittable based on local practice/guidelines.
2. As the aim is to prevent secondary injury by ensuring viable tissue is receiving an adequate amount of oxygen, the PbtO_2_ probe should be placed in a tissue region free of focal lesions on CT imaging, at a depth of approximately 2 cm from the cortical surface (the aim is to ‘sample’ subcortical white matter).
3. The probe should be sited wherever possible in non-eloquent tissue. The right frontal lobe is generally an ideal anatomical site, unless there is a contraindication such as craniotomy flap, compound depressed skull fracture, significant contusion or parenchymal injury on the right side, and then the left side should be used.
4. Ventriculostomy is optional for both cohorts throughout study enrolment. If an external ventricular drain is inserted, this can be sited contralateral or ipsilateral to study probes, according to the clinical judgement of the treating team.

If a craniectomy is performed (either at the same time or subsequently), the bolt may need to be positioned on the contralateral side or at an alternative site on the same side (eg, anteromedial to the craniectomy site) provided the principles outlined above are followed. The PbtO_2_ probe may also be placed via tunnelling under the scalp rather than through a bolt system. This may be the chosen insertion method at some sites or may be done in patients who require a craniectomy.

Following insertion of the PbtO_2_ catheter, a fractional inspired oxygen (FiO_2_) challenge is performed to assess sensor responsiveness. This challenge is considered successful if an increase of at least 5 mmHg in PbtO_2_ is observed. A failed FiO_2_ challenge may imply PbtO_2_ catheter malfunction and/or malposition (as indicated by CT scan), in which catheter replacement is recommended. Protocolised interventions are only commenced once probe reliability has been demonstrated.

Participating hospitals have the option to use ICM+ software (Cambridge Enterprise, Cambridge, UK) to continuously record ICP and PbtO_2_ measurements. For sites where the use of ICM+ is not feasible, PbtO_2_ measurements will be extracted from stored data in the monitoring device. This will be supplemented by ICP and PbtO_2_ data recorded in the medical record.

### Clinical management protocols

The baseline requirements for clinical care are outlined in Box 2. In addition, clinical management guidelines based on the Brain Trauma Foundation^4^ and Seattle International Severe Brain Injury Consensus Conference^19^ recommendations and reflecting best practice for the management of sTBI were drafted for all participants. Patients enrolled in both groups are managed according to these guidelines, which set out the tiers of physiological interventions available to treating clinicians for both PbtO_2_/ICP and ICP-guided management.

Box 2 **Baseline requirements for sTBI management**

**Lines/fluids**

- Arterial line and central line in place.
- Isotonic crystalloid preferred resuscitation fluid and avoid albumin.
- Cerebral perfusion pressure ≥60 mmHg.
- After initial resuscitation in otherwise stable patients, positive daily fluid balances >500 mL should be avoided.

**Serial evaluation of neurological status (including pupillary reactivity)**

**Patient position**

- Position 30°–45° head up, avoiding venous obstruction in the neck.

**Sedation**

- Adequate analgesia and sedation (to treat pain and agitation, independent of intracranial pressure); temperature management aim is core body temperature <38°C^­^.

**Haemoglobin**

- Aim haemoglobin >70 g/L.

**Glycaemic control**

- Aim normoglycaemia (as per local guidelines).

**Serum**

- Sodium aim for normonatraemia (Na+: 135–155 mmol/L).

**Prophylactic anti-seizure medication**

- Prophylactic anti-seizure medication for 7 days post sTBI. Consider electroencephalogram monitoring (where available).

sTBI, severe traumatic brain injury.

Interventions are to be commenced within 15 min of the start of a clinical episode (defined as an abnormality in ICP and/or PbtO_2_ lasting more than 5 min). The initial choice of treatment option from any tier is based on local preferences/practice and what is likely to be most effective in the clinical situation. If the abnormality does not resolve in a timely fashion, additional interventions within the same tier can be used or escalation to the next tier can be undertaken. In the event of no improvement in the abnormal parameter, escalation between tiers is mandated after a maximum of 60 min (figures2  3). Several inter-tier recommendations are also provided. The ICP target for each patient is based on local guidelines, typically being 20 mmHg or 22 mmHg.

The optimal duration of intracranial monitoring is unknown; however, a minimum period of 48 hours of protocolised care is mandatory. Cessation of study interventions within 48 hours of insertion of monitoring, in a patient who has not transitioned to end-of-life care, will constitute a protocol violation.

### Discontinuation of monitoring

Study interventions can be discontinued, and the patient considered ‘off protocol’, if any of the following conditions are met:

1. Recovery of neurological function (eg, Glasgow Coma Scale (GCS) motor score=6).
2. A specific indication to remove intracranial monitoring develops, such as bleeding or infection.
3. Inability to measure PbtO_2_ or ICP for >48 hours, due to technical issues.
4. Withdrawal of consent to participate in BONANZA-GT.
5. The occurrence of a serious adverse event (SAE) where, in the treating clinician’s opinion, the patient should be withdrawn from the study.
6. The treating clinical team elects to discontinue protocolised study interventions (after a minimum of 48 hours), so as to facilitate de-sedation and neurological assessment, or as per local guidelines.

Given existing clinical uncertainty, BONANZA-GT does not stipulate a maximum duration of intracranial monitoring, which remains at the discretion of the treating clinician, informed by local procedures and guidelines, and the manufacturer’s instructions. The following apply:

1. A minimum of 48 hours of protocolised care is required.
2. The use of any intracranial monitoring device must be consistent with local approvals.
3. Where the patient has been monitored for over 7 consecutive days, and otherwise the patient is stable (eg, stable neuroimaging), the focus should be on de-escalation of interventions.
4. ICU daily data collection is censored at 10 days.

### Study outcome measures

Details of all outcome measures are listed in Box 3.

Box 3 **Brain Oxygen Neuromonitoring in Australia and New Zealand Assessment – Global Trial primary and secondary outcome measures**

**Primary outcome measure**

- Proportion of favourable neurological outcomes (GOS-E: 5–8) at 6 months following injury.

**Secondary outcome measures**

- Probability of an equal or greater GOS-E level at 6 months compared with the probability of a lesser GOS-E level using a proportional odd model.
- Probability of an equal or greater GOS-E level at 6 months compared with the probability of a lesser GOS-E level using the ‘sliding dichotomy’ method.
- EQ-5D-5L at 6-month and 12-month post-injury.
- Proportion of favourable (GOS-E: 5–8) neurological outcomes at 12 months following injury.
- Mortality (all cause) at intensive care unit discharge, hospital discharge, and 90-days and 180-days post-injury.
- Incidence of adverse events.

GOS-E, Glasgow Outcome Scale-Extended.

### Data management and monitoring

A web-based database is used for data entry by trained staff at each study site. The BONANZA-GT database is also used for randomisation, screening and to create data queries, data validations and reports. Protocol deviations (defined as unanticipated or unintentional departure from the expected conduct of an approved study that is not consistent with the current research protocol or consent document) are also recorded in the database. For a list of predefined protocol deviations, please refer to the online supplemental material.

Data collection is restricted primarily to those variables necessary to define clinical patient characteristics, including baseline demographics, primary diagnoses, physiological parameters, diagnostic interventions, therapeutic interventions and documentation of deaths and other SAEs. All investigators and research staff will comply with the legislative requirements of their jurisdiction regarding the collection, storage, processing and disclosure of personal information. Confidentiality of all patient data will be maintained by the use of unique identifiers.

Overall data quality will be monitored by a study representative and include onsite monitoring visits, remote monitoring and central monitoring via the database. Data monitoring visits provide an opportunity to review and follow-up on any SAEs as well as to ensure that the consent procedures were followed according to local ethics requirements.

### Safety

An independent data and safety monitoring committee (DSMC), comprising experts in clinical trials, biostatistics, neurosurgery and intensive care medicine, was established before patient enrolment to review all trial protocols. The DSMC will assess the differential cumulative ‘SAE’ reports received after every 100 patients enrolled. In addition, after enrolling 430 patients (half of total recruitment), recruitment data, all safety-related data and outcome data will be reviewed by the DSMC in an un-blinded fashion. Consistent with previous literature,^20^ adverse events already defined and reported as study outcomes are not labelled and reported a second time as SAEs. The DSMC can, in its absolute discretion, request assessment of any other trial data at any time.

It is recognised that the study patient population will experience several common abnormalities in symptoms and clinical presentation due to the severity of the underlying disease and the impact of standard therapies. These do not necessarily constitute an adverse event unless they are directly related to study interventions, require significant intervention or are of concern in the investigator’s clinical judgement.

A SAE is defined as any adverse event that: results in death, is life-threatening, requires hospitalisation or prolongation of current hospitalisation, results in persistent or significant disability or incapacity, or is an important medical event which requires intervention to prevent one of the previously listed outcomes. For a list of predefined adverse clinical events, please refer to the online supplemental material.

### Statistical analyses

The proportion of participants in the control group with a favourable outcome (Glasgow Outcome Score-Extended (GOS-E) >4) at 6 months in the Decompressive Craniectomy in Diffuse Traumatic Brain Injury study was 49%.^21^ In the Erythropoietin in Traumatic Brain Injury study, in those with a severe head injury (GCS 3–8), 50% of patients in the control arm had a favourable outcome.^22^ We considered a clinically relevant effect size to be 10% absolute (20% relative) increase in favourable outcomes (a conservative estimate, given the OR for a favourable outcome was 1.8 in BOOST-2^17^). As such, with a control event rate of 50%, with 80% power, and a two-sided α of 0.05, a sample size of 816 patients is required. Based on a combined withdrawal and loss to follow-up rate of 5%, consistent with previous trials, we have inflated the sample size to 860 patients (430 in each group).

We prespecified 10% absolute improvement in favourable GOS-E at 6 months as clinically important. With an expected control rate of 50%, this equates to a number-needed-to-treat of 10, which reflects a meaningful gain in independent survival for patients and families. This threshold is consistent with absolute differences commonly targeted in phase III TBI trials. Furthermore, to quantify smaller distributional shifts in outcome, secondary analysis will be performed using GOS-E as an ordinal outcome.

Randomised patients from phases I and II are pooled for the primary efficacy analysis; run-in patients are non-randomised and excluded. The primary analysis will use a covariate-adjusted model, including site and covariates known to impact outcome, thus improving precision in the presence of between-patient and between-site heterogeneity. Analysis of patient outcomes will use an intention-to-treat principle and include all randomly assigned patients except those withdrawing consent for use of all trial data. Prior to the enrolment of the last patient, a detailed statistical analysis plan will be published separately.

## Ethics and dissemination

This study is undertaken in accordance with all relevant ethical, regulatory approvals and guidelines published by the National Health and Medical Research Council of Australia, the Declaration of Helsinki, its subsequent amendments, and the ICH‐GCP guidelines.

Ethics applications will be submitted to all relevant ethics boards in every jurisdiction that is participating. Inclusion of adults who lack the capacity to provide prospective informed consent will be governed in accordance with the legal requirements of each participating site. In Australia, the study was first approved by the Alfred Human Research Ethics Committee (ID: 393/19) on 23 October 2019. Country-specific ethics approvals were subsequently obtained from the following committees: Comité d'Ethique hospitalo Erasme – ULB (ID: P2022/351), Helsinki Ethics (ID:2296/2020), Comité de protection des personnes Ile de France (38RC23.0305), ETHIK-KOMMISSION Freiburg Germany (ID: 21–1554), Ethics (Medical Research) Committee Beaumont Hospital (ID: 22/46), Central Health and Disability Ethics Committee (NZ, ID: 20/CEN/204), Bern Ethics Committee (ID: 2020–01111), East of England - Cambridge Central Research Ethics Committee (ID: 21/EE/0216) and Dictamen Del Comité De Ética De La Investigación (Spain, ID: 24/346).

Patients who are incapable of giving consent in emergency situations are an established exception to the general rule of informed consent in clinical trials and acknowledged in the Declaration of Helsinki. Patients with sTBI present at all hours under emergency conditions and often the legally authorised representative is not known. Furthermore, due to the time-critical nature of the necessary treatment, it is expected in most cases that there will not be sufficient time to discuss the research in a manner that will allow free and informed consent with the designated legally authorised decision-maker.

As soon as practicable following recruitment, research staff will provide the legal representative or the patient’s family with full verbal explanation of the research, including the option to withdraw from the study if they wish, in addition to printed information for their consideration. Should participants regain capacity while they are in the hospital, they will be provided with information, and consent to continue in the trial will be sought from them.

The results from this trial will be presented in national and international conferences. The principal publication from the study will be in the name of the BONANZA-GT Investigators, with full credit assigned to all collaborating investigators, research coordinators and institutions. Where individual names are required for journal publication, it will be that of the writing committee, with the chairs of the writing committee listed first and last, and subsequent authors listed alphabetically. Authorship using the name of these individuals will be granted using the International Committee of Medical Journal Editors definitions and depending on personal involvement and fulfilment of the respective roles of authors.

## Discussion

Despite the enormous costs associated with sTBI, outcomes are not improving. Clinical management strategies remain primarily based on expert opinion and observational studies due to a lack of robust high-quality evidence in the field. Indeed, several of the limited number of high-quality randomised controlled trials in sTBI have provided conclusions that challenge long-standing assumptions and fundamental principles of management.^21 23^ Multimodality monitoring of parameters such as ICP, PbtO_2_ and electroencephalogram has been adopted to varying degrees in tertiary care centres in the USA and Europe. Despite the enormous potential of these tools to optimise clinical management, rigorous evaluation of clinical benefit has not taken place. Without adequately powered randomised trials, there is a risk that ineffective modalities may become widely adopted (resulting in a loss of equipoise for a future trial), and a parallel risk that effective modalities do not become widely implemented due to a lack of high-quality evidence. At present, there is limited phase III data exploring the impact of PbtO_2_-based clinical management in sTBI. As such, BONANZA-GT will provide urgently needed evidence evaluating the role of this technology in routine clinical care.

### Risk of intracranial monitoring

Intracranial neuromonitoring involves an invasive procedure that is not risk-free. Current literature indicates a rate of probe-associated bleeding between 2.6% and 11%^24^,^26^ and the OXY-TC trial found a small but significant increase in the rate of intracerebral haematoma in the ICP/PbtO_2_ group (4%), compared with the ICP-only group (0%).^18^ BONANZA-GT aims to minimise patient risk by recruiting only patients with a clearly documented requirement for intracranial monitoring which is independent of trial inclusion. Phase III of BONANZA-GT now only requires PbtO_2_ catheter placement in patients allocated to the intervention arm and avoids any potential unnecessary risk of PbtO_2_ probe-related complications in control patients. All study devices must be inserted by trained neurosurgical staff and monitored carefully for any associated adverse events. The BONANZA-GT DSMC will assess all device-related adverse events and provide independent advice regarding any safety signal.

### Risks associated with treatment algorithm

Treatment of ICP elevations in the BONANZA-GT protocol is based on widely accepted management protocols. The available evidence indicates that the risks of these interventions are outweighed by the potential for decreased ICP-related mortality and morbidity.^4 19^ Participants who are randomised to the ICP-only treatment arm will not be subject to any risks over and above those associated with standard care. Participants randomised to PbtO_2_-guided management may be exposed to the risk of specific treatment-related complications from interventions such as fluid boluses or vasopressors to increase CPP,^27^ transfusion of packed red blood cells^28^ and ventilator adjustments.^29^ It is important to note that these interventions are effective at reversing cerebral hypoxia and are not associated with significant adverse events in prior literature (including the recent OXY-TC trial). In addition, all these interventions are commonly provided in the setting of acute trauma resuscitation and management. More specifically, recently published trials support a liberal red blood cell transfusion strategy in sTBI patients,^30 31^ and it is possible BONANZA-GT may provide supporting evidence as to physiological mechanisms underlying the beneficial effects observed in these studies.

### Benefits

The BOOST-2 trial was stopped early by the DSMC for efficacy in the primary outcome—a reduction in brain tissue hypoxia. A signal towards clinical benefit was also reported, but the trial lacked the required statistical power to support any definitive conclusion. Conversely, the more recent OXY-TC trial reported a neutral result, with no significant benefit associated with PbtO_2_-guided management post sTBI. While this result is noteworthy, the authors reported an imprecise effect estimate (OR 0.9, 95% CI 0.6 to 1.6) which could indicate both significant harm and benefit. The potential benefit of PbtO_2_-guided management therefore remains uncertain in this patient cohort.

### Limitations

It is important to note that PbtO_2_ monitoring requires significant resource allocation. As such, our study findings will be most relevant to patients in well-resourced health care systems able to support high-frequency neuromonitoring in the ICU. Validity may not extend to less well-resourced healthcare systems in low-income and middle-income countries. We contend, however, that our findings will advance our overall understanding of the pathophysiology of sTBI and provide insights into how best to manage this pathology in all contexts.

In conclusion, BONANZA-GT will assess the role of PbtO_2_ optimisation in patients with sTBI. It is sufficiently powered to detect statistically significant differences in the primary outcome between the control and intervention groups. This should provide the required evidence to support or refute the role of this technology and the optimisation of brain tissue oxygenation in the care of sTBI patients.

## Supplementary material

10.1136/bmjopen-2025-106962 online supplemental file 1
