# Supplementary material for: Brain tissue oxygen monitoring for severe traumatic brain injury: the international multicentre randomised controlled BONANZA-GT study protocol
Source: BMJ Open. 2025 Oct 2;15(10):e106962. doi: 10.1136/bmjopen-2025-106962 (PMC12496108; doi:10.1136/bmjopen-2025-106962)
Supplement: online supplemental file 1 [file bmjopen-15-10-s001.docx]

**Brain tissue oxygen monitoring for severe traumatic brain injury: the international multicentre randomised controlled BONANZA-GT study protocol**

**Supplemental Material**

1. List of predefined protocol deviations:

- Patient randomised but not eligible
- Patient randomised but study intervention not administered
- Intracranial pressure (ICP) > target for > 30 minutes with no attempt to implement the BONANZA-GT algorithm
- ICP > 25mmHg > 30 minutes with no attempt to implement the BONANZA-GT algorithm
- The brain tissue oxygen (PbtO_2_) was 15-19 mmHg for > 30 minutes with no attempt to implement the BONANZA-GT algorithm
- The PbtO_2_ was < 15 mmHg for > 30 minutes with no attempt to implement the BONANZA-GT algorithm
- Placement of devices more than 24-hours from the time of injury
- Less than 48 hours of mandatory BONANZA-GT protocolised care
- Other

1. Clinical adverse events:

- Acute Myocardial Infarction
- New Onset left ventricular dysfunction
- Acute Respiratory Distress Syndrome/Acute Lung Injury
- Central Nervous System Infection
- Ventriculitis
- Meningitis
- Cerebral abscess
- Transfusion reaction Not Further Specified (NFS)
- Acute haemolytic reaction
- Non haemolytic reaction
- Allergic reaction
- Transfusion transmitted bacterial infection

Probe related adverse events:

- Probe complication
- Probe complication requiring surgical intervention
- Significant probe related complication, but surgical intervention is not required
- Probe failure or malfunction
