# Supplementary material for: Brain tissue oxygen monitoring for severe traumatic brain injury: the international multicentre randomised controlled BONANZA-GT study protocol
Source: BMJ Open. 2025 Oct 2;15(10):e106962. doi: 10.1136/bmjopen-2025-106962 (PMC12496108; doi:10.1136/bmjopen-2025-106962)
Supplement: Supplementary file 2 [file bmjopen-2025-106962.reviewer_comments.pdf]

PEER REVIEW HISTORY

BMJ Open publishes all reviews undertaken for accepted manuscripts. Reviewers are asked to complete a checklist review form and are provided with free text boxes to elaborate on their assessment. These free text comments are reproduced below.

ARTICLE DETAILS

Title (Provisional)

Brain tissue oxygen monitoring for severe traumatic brain injury: the international multicentre randomised controlled BONANZA-GT study protocol

Authors

Udy, Andrew Alexander; Jeffcote, Toby; Battistuzzo, Camila R; Sharry, Aideen; Adamides, Alexios A; Ainscough, Kate; Alliegro, Patricia V; Anstey, James; Bailey, Michael; Barea-Mendoza, Jesús Abelardo; Bellapart, Judith; Bouzat, Pierre; Byrne, Kathleen; Delaney, Anthony; Drummond, Katharine J.; Haenggi, Matthias; Hays, Leanne M; Higgins, Alisa; Huet, Olivier; Hunn, Martin K; Jakob, Stephan M; Lewis, Philip M; Lu, Kuan-Ying; Menon, David; Moore, James; Murray, Patrick T; Porter, John; Reddi, Benjamin; Rosenfeld, Jeffrey V; Taylor, Jonathon D; Trapani, Anthony; Vallance, Shirley; Wolf, Stefan; Payen, Jean-Francois; Taccone, Fabio Silvio; Nichol, Alistair; Cooper, D James

---

VERSION 1 - REVIEW

**Reviewer**	**1**
**Name**	**Morris, Meg**
**Affiliation**	**La Trobe Univ, Room 423 HS3**
**Date**	**29-Aug-2025**
**COI**	**None**

This is a well written, scholarly and engaging manuscript on an important topic - a protocol to examine optimal methods for brain oxygen monitoring in patients with severe brain injury. Some revisions are required:

1. The introduction is clear yet is insufficiently referenced. Especially in the first paragraph and throughout please cite recent evidence-based research to support the arguments being made.

2. A major limitation is that the reference list seems very out of date. Please can the research team do a new search of PubMed and Google Scholar (and other relevant databases) and add many more of the articles that have been published since 2022. There

are lots of relevant articles and it is really unclear why they have not been cited. All authors are advised to review the references in the revised manuscript to check its up to date.

3. The sample sizes for the two groups seem modest. Please can the statistician recheck the sample size calculations and confirm that the three-phase design has been taken into account in the ss calculations. Also please recheck the variability between patients that you have factored in - these patients are highly variable in their signs and symptoms. Update the ss calculations if needed.

4. The trial is powered to detect a 10% absolute difference in the primary outcome

(GOS-E at 6-months post injury) between the control and intervention groups. Please justify in the manuscript that this will be a clinically significant difference.

5. In the Methods section please add more details on how informed consent will be gained given that patients have brain injuries.

6. The DSMC will assess the differential cumulative "serious adverse event" reports received after every 100 patients enrolled. Should the DSMC also be informed of serious adverse events (especially death) within 24 hours of when they actually occur? Please look at GCP training guidelines on when DSMC and other parties should be notified of adverse events.

7. In the Discussion please write 2-3 more sentences on the limitations of this trial, including the generalizability and extent to which it controls for people from different cultures, languages and geographical regions.

8. In the Ethics statement, please give the name and ethics approval number for the central ethics committee for this trial.

9. In the Supplementary files please spell out abbreviations other than ICP and Bonanza (such as NFS)

VERSION 1 - AUTHOR RESPONSE

Reviewer: 1
Prof. Meg Morris, La Trobe Univ
Comments to the Author:

This is a well written, scholarly and engaging manuscript on an important topic - a protocol to examine optimal methods for brain oxygen monitoring in patients with severe brain injury. Some revisions are required:

1. The introduction is clear yet is insufficiently referenced. Especially in the first paragraph and throughout please cite recent evidence-based research to support the arguments being made.
RESPONSE: Thank you for pointing this out – we have updated the references in the first

paragraph to more contemporary evidence.
2. A major limitation is that the reference list seems very out of date. Please can the research team do a new search of PubMed and Google Scholar (and other relevant databases) and add many more of the articles that have been published since 2022. There are lots of relevant articles and it is really unclear why they have not been cited. All authors are advised to review the references in the revised manuscript to check its up to date.
RESPONSE: Thank you for the comment – where possible we have updated the references to more recent evidence. In several cases we refer to more foundational evidence in the field of PBtO2 monitoring which is necessarily older.

3. The sample sizes for the two groups seem modest. Please can the statistician recheck the sample size calculations and confirm that the three-phase design has been taken into account in the ss calculations. Also please recheck the variability between patients that you have factored in - these patients are highly variable in their signs and symptoms. Update the ss calculations if needed.
RESPONSE: Thank you. We have rechecked the sample-size calculations and confirmed that the design and between-patient variability have been handled appropriately.
Three-phase design: The run-in patients are non-randomised and excluded from efficacy analyses, as stated in the Trial design section. The primary efficacy analysis pools all randomised patients from Phases 1 and 2 (intention-to-treat). This structure has been used in the power calculation because power depends on the total randomised N; the run-in contributes no randomised patients and therefore does not affect the required sample.
Sample-size assumptions: As reported, we powered for a 10% absolute improvement in favourable GOS-E at 6 months (control 50%), 80% power, two-sided p=0.05, and 5% attrition, giving N=860 (430/arm). We have re-run the calculation under these inputs and confirm no change is required.
Between-patient variability: We acknowledge the clinical heterogeneity in severe TBI. The primary analysis will apply an intention-to-treat framework and (as clarified in the revised Statistical analyses paragraph) a covariate-adjusted model including site and known covariates for outcome will be performed which improves precision and mitigates between-site and between-patient variability. For increased transparency, we have added the following details:
Statistical analyses
Analysis of patient outcomes will employ an intention-to-treat principle and include all randomly assigned patients except those withdrawing consent for use of all trial data. "Randomised patients from Phases 1 and 2 are pooled for the primary efficacy analysis; run-in patients are non-randomised and excluded. The primary analysis will employ a covariate-adjusted model including site and covariates known to impact outcome, thus improving precision in the presence of between-patient and between-site

heterogeneity"

4. The trial is powered to detect a 10% absolute difference in the primary outcome (GOS-E at 6-months post injury) between the control and intervention groups. Please justify in the manuscript that this will be a clinically significant difference.
RESPONSE: Thank you. We agree that the clinical meaning of the target effect should be explicit. We have added a justification to the Sample size section. A 10% absolute increase in the proportion with favourable GOS-E at 6 months corresponds to a number-needed-to-treat of 10 when the control rate is 50%. For survivors of severe TBI, this magnitude represents one additional patient achieving independence for every ten treated, which our clinician and consumer advisors considered a meaningful benefit. The chosen effect size is also consistent with absolute differences commonly targeted in phase 3 TBI trials. We have retained the planned ordinal and utility-focused secondary analyses to capture smaller but still important shifts in outcome.
“We prespecified a 10% absolute improvement in favourable GOS-E at 6 months as clinically important. With an expected control rate of 50%, this equates to a number-needed-to-treat of 10, which reflects a meaningful gain in independent survival for patients and families. This threshold is consistent with absolute differences commonly targeted in phase 3 TBI trials. Furthermore, to quantify smaller distributional shifts in outcome, secondary analysis will be performed using GOS-E as an ordinal outcome.

5. In the Methods section please add more details on how informed consent will be gained given that patients have brain injuries.
RESPONSE: Thank you for the comment – we give a detailed breakdown of the consent process in the ethics section and have added a sentence to the methods section directing the reader to the ethics section for clarity.
‘Given that patients will lack the ability to provide informed consent at the time recruitment, inclusion in the study will be on the basis of a deferred consent model (see Ethics section for further details).’

6. The DSMC will assess the differential cumulative “serious adverse event” reports received after every 100 patients enrolled. Should the DSMC also be informed of serious adverse events (especially death) within 24 hours of when they actually occur? Please look at GCP training guidelines on when DSMC and other parties should be notified of adverse events.
RESPONSE: Thank you for this comment. The GCP guidelines mandate immediate reporting of serious adverse events by investigators to the Sponsor followed by written documentation. There is however, no explicit guidance around the timing of reporting of SAEs by the sponsor to the DSMC and sponsor discretion is applied to the reporting of SAEs to the DSCMC. The current guidance is that serious adverse events should be

reported to the DSMC at pre-specified review points e.g. after every 100 patients, or those time points set out in the study protocol. In practice, for any device related SAE's, the BONANZA-GT Management Committee has made a decision to forward this information to the DSMC, as soon as complete documentation has been received by Sponsor.

7. In the Discussion please write 2-3 more sentences on the limitations of this trial, including the generalizability and extent to which it controls for people from different cultures, languages and geographical regions.
RESPONSE: Thank you for this important point, we have added a short paragraph to address this limitation.
Limitations
It is important to note that PbtO2 monitoring requires significant resource allocation. As such our study findings will be most relevant to patients in well resourced health care systems able to support high frequency neuro-monitoring in the ICU. Validity may not extend to less well resourced healthcare systems in low and middle income countries. We contend however that our findings will advance our overall understanding of the pathophysiology of sTBI and provide insights into how best to manage this pathology in all contexts.

8. In the Ethics statement, please give the name and ethics approval number for the central ethics committee for this trial.
RESPONSE: As BONANZA-GT is an international multi-centre randomised controlled trial, there is no single HREC which has jurisdiction across all regions where the trial is being performed. Within each country, separate ethical and regulatory requirements have been adhered to. As such, in the manuscript, we provide the details of where the study was first approved, but note this is not a "central" Ethics committee.

9. In the Supplementary files please spell out abbreviations other than ICP and Bonanza (such as NFS)
RESPONSE: Thank-you. This has been actioned.

Reviewer: 1
If you have selected 'Yes' above, please provide details of any competing interests.: Not applicable

---

VERSION 2 - REVIEW

**Reviewer**	**1**
**Name**	**Morris, Meg**

Affiliation La Trobe Univ, Room 423 HS3

Date 09-Sep-2025

COI

The authors have done a very good job of addressing all of the feedback that I raised.
